# The contribution of microbially produced nanoparticles to sustainable development goals

**DOI:** 10.1111/1751-7915.12788

**Published:** 2017-08-03

**Authors:** Miguel E. Cueva, Louise E. Horsfall

**Affiliations:** ^1^ SynthSys, CSEC and School of Biological Sciences University of Edinburgh Edinburgh UK

## Abstract

Nanoparticles (NPs), particles having one or more dimensions below 100 nm, are currently being synthesized through chemical and physical methods on an industrial scale. However, these methods for the synthesis of NPs do not fit with sustainable development goals. NP synthesis, through chemical and physical methods, requires high temperatures and/or pressures resulting in high energy consumption and the generation of large amounts of waste. In recent years, research into the synthesis of NPs has shifted to more green and biological methods, often using microorganisms. A biological approach has many advantages over chemical and physical methods. Reactions are catalysed in aqueous solutions at standard temperature and pressure (cost effective and low energy syntheses). This method does not require solvents or harmful chemicals, making NP biosynthesis a greener and more eco‐friendly method. Furthermore, NP synthesis by microbes does not require the use of pure starting materials; thus it can simultaneously be used for the bioremediation of contaminated water, land and waste, and the biosynthesis of NPs. Therefore the biosynthesis of NPs contributes to the sustainable development goals, while the alternative physical and chemical methods exclusively utilize scarce and expensive resources for NP synthesis.

## Abstract 2

Nanoparticles are currently being synthesized through chemical and physical methods on an industrial scale. However, these methods of synthesis do not fit with sustainable development goals as they require high temperatures and/or pressures resulting in high energy consumption and the generation of large amounts of waste. In recent years, research into the synthesis of NPs has shifted to more green and biological methods, often using microorganisms.

Nanoparticles (NPs), particles having one or more dimensions below 100 nm, are currently being synthesized through chemical and physical methods on an industrial scale. However, these methods for the synthesis of NPs do not fit with sustainable development goals. NP synthesis, through chemical and physical methods, requires high temperatures and/or pressures resulting in high energy consumption and the generation of large amounts of waste. In recent years, research into the synthesis of NPs has shifted to more green and biological methods, often using microorganisms. A biological approach has many advantages over chemical and physical methods. Reactions are catalysed in aqueous solutions at standard temperature and pressure (cost‐effective and low‐energy syntheses). This method does not require solvents or harmful chemicals, making NP biosynthesis a greener and more eco‐friendly method. Furthermore, NP synthesis by microbes does not require the use of pure starting materials; thus, it can simultaneously be used for the bioremediation of contaminated water, land and waste and the biosynthesis of NPs (Pollman *et al*., 2006; Macaskie *et al*., 2010). Therefore, the biosynthesis of NPs contributes to the sustainable development goals, while the alternative physical and chemical methods exclusively utilize scarce and expensive resources for NP synthesis.

Biogenic metal nanoparticles are produced by microorganisms utilizing their natural metal resistance and metabolic pathways, through either an intracellular or an extracellular mechanism. The intracellular mechanism involves ion transportation into the microbial cell where they are then reduced to their elemental form through electrostatic and enzymatic interactions, forming NPs that can be held within the cell. The extracellular mechanism is mediated either through enzymatic reduction at the cell surface or by secreted molecules that reduce metal ions into their elemental form (Hulkoti and Taranath, 2014).

Potential applications of biogenic metal NPs range from various biomedical purposes (e.g. antiviral, antibacterial, antiparasite, medical imaging, drug delivery, cancer treatment and medical diagnostics), through environmental remediation purposes (e.g. contaminant/pollutant degradation and catalytic treatments of aqueous organic compounds), to industrial purposes (e.g. enhance fuel cell performance, catalytic organic synthesis and nanoelectrochemistry) (Schröfel *et al*., 2014). While research using biogenic nanoparticles is relatively new, researchers are testing them for potential applications in biomedical contexts (anticancer, drug delivery and antimicrobial). For instance, AgNPs produced by *Bacillus licheniformis* have the potential for use as an antiagiogenic (inhibit blood vessel formation to prevent the systemic spread of cancer cells) (Kalishwaralal *et al*., 2009), while AgNPs produced by *Fusarium oxyporum* have been incorporated into textile fabrics to inhibit or prevent infections from pathogenic bacteria (Durán *et al*., 2007). Nanomedicine is a growing scientific field and has tremendous prospects for the improvement of the diagnosis and treatment of human diseases, where biogenic NPs will not only be applied, but their production will also fit within sustainable development goals.

Some examples of microorganisms that are capable of synthesizing NPs are as follows: *Desulfovibrio desulfuricans*,* Cupriavidus metallidurans*,* Bacillus subtilis*,* Escherichia coli*,* Rhodococcus* sp*., Candida glabrata* and *Verticillium* sp. (Park *et al*., 2015). Currently, the range of metals that are able to be produced in nanoparticle form by microbes include (but is not limited to) As, Ag, Al, Au, Cd, Cr, Cu, As, Pb, Pd and Pt (Pantidos and Horsfall, 2014).

Although microbially produced metal NPs have many potential applications, there are still many drawbacks and problems to be addressed in their synthesis. First, biogenic metal NPs may be produced with a broad morphology and size range. Second, their synthesis may not result in sufficiently high yields and, depending on the microorganism used, it may be slow. Third, metal NPs are sometimes synthesized in unstable forms with reduced catalytic activities, and finally, the use of impure starting materials (e.g. waste) affects its downstream purification. In an effort to address these problems, synthetic biology, nanobiotechnology and genetic engineering are being researched to improve NP synthesis. Once size and morphology are controlled, yield is increased and downstream purification enhanced; the overall production of NPs will be both cost‐effective and greener (Edmundson *et al*., 2014).

Synthetic biology involves the design and engineering of microorganisms to make them capable of performing novel functions for industry, medicine and scientific research. Within the different applications of synthetic biology are those exploring the potential biosynthesis of metal NPs and will utilize its modular genetic engineering approach to enhance NP quality and quantity.

Research with cell surface engineering for the synthesis of metal NPs has been carried out through the heterologous expression of synthetic chelatins (polypeptide chelators rich in metal binding amino acids, e.g. EC20), phytochelatins and metallothionein (cysteine‐rich polypeptide chelators found in plants) and phytochelatin synthase (the enzyme responsible for synthesizing phytochelatins from glutathione). An early synthetic biology study successfully synthesized Au, Ag, Fe, Te, CdZn, CdSe, CdTe, ZnSe, CdCs, PrGd, SrGd, SrPr, FeAg, FeCo, FeMn, CdSeZn, FeCoNi, FeCoMn, CdSeZnTe and AuCdSeZn NPs with a recombinant *E. coli* strain co‐expressing phytochelatin synthase and metallothionein (Park *et al*., 2010). While more recently the *in vivo* synthesis of EuSe NPs was performed using recombinant *E. coli* cells expressing phytochelatin synthase, phytochelatins and metallothionein, these EuSe NPs exhibited high fluorescence intensities and strong magnetic properties, and their anticancer properties were demonstrated effectively (Kim *et al*., 2016).

The overexpression of key proteins and enzymes from the NP synthesis pathway can enhance a microorganism's ability to sequester metals and significantly increase their tolerance to metal concentrations. A recent study produced a recombinant *E. coli* strain containing arsenic resistance genes from a number of sources, and it was then demonstrated that the engineered *E. coli* is able to not only tolerate high levels of arsenic, but is also able to convert the arsenic to an insoluble form, thereby removing significant amounts of arsenic from a solution (Edmundson and Horsfall, 2015).

The use of encapsulins also has promising applications within nanobiotechnology. The ability to engineer cellular compartments and functional nanoarchitectures under environmentally sustainable conditions has been fruitful for the synthesis of monodispersed NPs. A recent study engineered the *Thermotoga maritima* encapsulin to yield a designed compartment for the size‐constrained synthesis of Ag NPs. Such NPs exhibited a uniform shape and size distribution as well as long‐term stability. When their antimicrobial activity was tested, it proved to be superior to that of silver nitrate and citrate‐capped Ag NPs (Giessen and Silver, 2016). Another promising application of nanobiotechnology for the synthesis of monodispersed NPs is through the use of *Dps* proteins, oligomeric cage‐like molecules that contain ferroxidase centres with iron oxidation/storage capacity. Research on *Dps* proteins from starved *Listeria innocua* cells, and its mutant (lacking catalytic ferroxidase centre) showed they can produce magnetite (Fe_3_O_4_) NPs, homogeneous NPs with an average diameter of 3 nm inside both non‐mutant and mutant cavities (Ceci *et al*., 2010). An alternative method for the synthesis of magnetite is through the biomineralization of iron with bacterial organelles called magnetosomes. Mms6, a magnetosome protein produced by the magnetotactic bacterium *Magnetospirillum magneticum* AMB‐1, has been shown to form magnetite NPs *in vitro*. Recent work which fused Mms6 onto microcontact‐printed assembled monolayer surfaces showed control of the formation and location of magnetite NP production in microscale arrays, thus creating magnetic NP patterns (Bird *et al*., 2016).

Demands for metal NPs have increased as the number of potential applications has grown. But it is hard to cheaply mass‐produce homogenous metal NPs, and no method for the production of biogenic metal NPs has been commercialized. However, recent studies have proved that the scaled‐up production of metal NPs, and therefore its future commercialization, is a real possibility (Moon *et al*., 2010; Byrne *et al*., 2015). The production of magnetite nanoparticles by the Fe(III)‐reducing bacterium *Geobacter sulfurreducens* has successfully been scaled up from the laboratory to pilot plant. The plant‐scale production produced up to 120 g of biomagnetite, with a maintained size distribution between 10 and 15 nm, and unchanged surface reactivity and magnetic properties (Byrne *et al*., 2015). Another recent study illustrating the potential for the commercialization of biogenic NP synthesis performed scale‐up experiments with *Thermoanaerobacter sp*. TOR‐39 (anaerobe Fe(III)‐reducing bacteria), using a 35 l reactor batch fermentation to obtain a yield of over 1 kg (wet weight) of magnetite in less than a month (Moon *et al*., 2010). Three key aspects for the mass production of NPs through a bacterial fermentation were elucidated in these studies: first, the rate at which particles are produced; second, the size reproducibility (monodispersed NPs); and finally, the ease of downstream recovery. However, the ultimate determinant as to the success of industrial nanoparticles biosynthesis will be the quality of the microbially produced NPs as compared to those produced chemically (Delay and Frimmel, 2012).

The future of microbially produced NPs is likely to involve an increase in the range of metals that microbes can produce NPs from, perhaps to even include nanoparticles made of metals which may not have a chemically derived counterpart. To achieve the aforementioned, the screening of metal NP synthesis from different microorganisms needs to be conducted. Such an approach identified the CuNPs synthesized by *Morganella morganii*, which are far more stable than their commercial counterpart (Ramanathan *et al*., 2013). A microbial process to produce such NPs on an industrial scale could hypothetically cost a fraction of what a traditional chemical synthesis would cost. If an adequate design of a large‐scale bioreactor configuration for the optimization of NPs synthesis is reached and recovery methods are perfected, the future industrial production of biogenic metal NPs seems like a real possibility. Not only would the mass production of biogenic metal NPs be of importance to the scientific world, but it could potentially benefit our society and the environment, generating wealth through the clean‐up of metal‐contaminated land, water and waste sites and obtaining a final product (metal NPs) that can be reintroduced into the world's economy.

## Conflict of interest

None declared.
